# Proteomic and metabolomic profiling of methicillin-resistant *Staphylococcus aureus* associated with invasive vs. non-invasive infections: uncovering key biomarkers and pathogenic pathways

**DOI:** 10.3389/fmicb.2026.1798070

**Published:** 2026-05-06

**Authors:** Syrine Boucherabine, Alexander D. Giddey, Rania Nassar, Lobna Mohamed, Subham Verma, Nelson C. Soares, Abiola Senok

**Affiliations:** 1 College of Medicine, Mohammed Bin Rashid University of Medicine and Health Sciences, Dubai, United Arab Emirates; 2 Department of Biological Sciences, Khalifa University, Abu Dhabi, United Arab Emirates; 3 Center for Applied and Translational Genomics, Mohammed Bin Rashid University of Medicine and Health Sciences, Dubai, United Arab Emirates; 4 Laboratory of Proteomics, Department of Human Genetics, National Institute of Health Doutor Ricardo Jorge (INSA), Lisbon, Portugal; 5 Comprehensive Health Research Centre (CHRC), NOVA Medical School, University NOVA of Lisbon, Lisbon, Portugal; 6 School of Dentistry, Cardiff University, Cardiff, United Kingdom

**Keywords:** invasive, metabolomic, methicillin resistant *Staphylococcus aureus*, MRSA, non-invasive, proteomics

## Abstract

**Introduction:**

Understanding the behavioral differences between invasive and non-invasive methicillin-resistant *Staphylococcus aureus* (MRSA) is essential for unraveling infection mechanisms and identifying biomarkers with translational potential. This study compared the proteomic and metabolomic profiles of MRSA isolates from diverse clinical presentations to uncover distinct molecular signatures.

**Methods:**

Invasive isolates were obtained from blood cultures (*n* = 23), while non-invasive isolates were derived from superficial skin infections (*n* = 49) and nasal colonizers (*n* = 24) from screening swabs. Proteins and metabolites were simultaneously extracted using a dual-phase methanol-based protocol. Proteomic analysis was performed on the Orbitrap Exploris 480, while metabolites were characterized using a TimsTOF mass spectrometer with an Apollo II electrospray ionization source. Data-independent acquisition (DIA) was applied, with peptide assignment carried out in DIA-NN and metabolite analysis using MetaboScape® 4.0.

**Results:**

Across all isolates, 2,000 proteins and 150 metabolites were identified. Comparative analysis revealed that invasive isolates exhibited consistently higher levels of two metabolites (sphinganine and phosphoserine) and one protein (staphylococcal secretory antigen SsaA2) compared to non-invasive. In contrast, three metabolites (cytidine, benzoic acid, and guanosine) and two proteins (small ribosomal subunit protein bS20 and bifunctional autolysin) were significantly reduced in invasive isolates. These findings highlight key molecular differences underpinning invasive potential in MRSA, providing insights into candidate diagnostic and therapeutic biomarkers. These findings highlight critical biological differences between invasive and non-invasive MRSA, offering valuable insights into potential diagnostic and therapeutic biomarkers.

## Introduction

Methicillin-resistant *Staphylococcus aureus* (MRSA) continues to pose a major global health challenge, causing both healthcare-associated and community-acquired infections (Turner et al., 2019). MRSA is capable of producing a wide spectrum of clinical manifestations, ranging from mild nasal colonization to severe invasive infections such as bacteremia, sepsis, and endocarditis (Lowy, 1998; Yamamoto et al., 2013). The ability of MRSA to switch between colonizing and invasive phenotypes underscores its remarkable adaptability and raises important questions about the molecular determinants that drive invasiveness. Despite extensive genomic studies on MRSA, the biological mechanisms that differentiate invasive from non-invasive isolates remain incompletely understood.

In recent years, omics-based approaches have provided deeper insights into MRSA pathogene-sis. Whole-genome sequencing (WGS) studies have delineated clonal diversity, identified resistance determinants, and traced the global spread of epidemic clones (Harris et al., 2010; Senok et al., 2020; Boucherabine et al., 2025). However, genomics alone does not fully capture the functional state of bacterial cells. Proteomics and metabolomics, by directly profiling proteins and metabolites, offer complementary perspectives that reflect dynamic changes in bacteri-al physiology, virulence, and metabolic adaptation (Khodadadi et al., 2020; Kondori et al., 2021; Lee et al., 2021; Torres-Sangiao et al., 2022; Tsuchida et al., 2020). Multi-omics integration is therefore a powerful approach to unravel how MRSA adapts to host environments and progresses from colonization to invasive disease.

Several studies have investigated the proteomes and metabolomes of *S. aureus* under specific experimental conditions, such as antibiotic stress or nutrient limitation, and have linked metabolic shifts to virulence regulation (Resch et al., 2006; Liu et al., 2014; Xu et al., 2020; Liebeke et al., 2011; Atshan et al., 2015; Rahman et al., 2022; Sulaiman et al., 2021). Yet, there is a critical gap in functional characterization of MRSA isolates, namely direct comparisons of clinical MRSA isolates stratified by invasive versus non-invasive phenotypes. Understanding how invasive and non-invasive MRSA differ in their proteomic and metabolic landscapes could provide valuable insights into molecular pathways that facilitate tissue invasion, immune evasion, and persistence.

In this study, we performed an integrative proteo-metabolomic analysis of MRSA isolates obtained from blood (representing invasive disease) and from wound and nasal specimens (representing non-invasive disease). By combining high-resolution label-free quantitative proteomics with untargeted metabolomics, we sought to identify molecular signatures and pathways that distinguish invasive from non-invasive isolates. This approach provides a functional layer of evidence complementing genomic data and has the potential to uncover biomarkers of invasive-ness, inform therapeutic strategies, and guide precision surveillance of MRSA in the region.

## Methods

### Bacterial isolates

A selection of 96 MRSA clinical isolates were investigated based on the isolate source. These included invasive isolates (*n* = 23) which were obtained from blood cultures of patients with bloodstream infections as well as non-invasive isolates (*n* = 49) which were identified from swabs of superficial skin infections of patients from the outpatient setting. In addition, nasal colonizer isolates (*n* = 24) which were obtained from screening anterior nares swabs and isolated during the same collection period were included. All isolates included in this study had been previously characterized using (VITEK) and DNA microarray analysis, confirming the presence of the *mecA* gene, consistent with MRSA classification (Boucherabine et al., 2025). The isolates were sub-cultured overnight on Columbia Blood Agar and bacterial pellet was prepared as previously described (Boucherabine et al., 2024).

### Protein and metabolite extraction

Protein and metabolite extraction was performed using the dual-functionality method (Boucherabine et al., 2024). Briefly, metabolite extraction was carried out by resuspending cell pellet in 300 μL of cold methanol and keeping it at −20 °C for 2 h. Samples were then flash frozen in liquid nitrogen and sonicated in a water bath sonicator. Samples were centrifuged to allow for the cell debris and proteins to pellet. The supernatant containing metabolites was transferred to a new labeled microcentrifuge tube and dried using speed vac at 38 °C and stored in −80 °C pending analysis. The cell pellet was used for protein extraction by resuspending it in the urea containing lysis solution (8 M Urea, 50 mM Tris–HCL) (pH 8). The samples were centrifuged and the supernatant was moved to a new labeled microcentrifuge tube, while the remaining pellet containing the cellular debris was discarded. The conserved supernatant containing the proteins extracted was then quantified using the modified Bradford quantification method (Ramagli and Rodriguez, 1985).

### Tryptic digestion

Tryptic digestion was carried out for the protein extracted. We prepared solutions containing 20 μg of extracted protein which were incubated with 1 mM dithiothreitol (DTT) for 1 h with gentle shaking at room temperature to break intra- and inter-protein disulfide bonds. This was followed by alkylation with 50 mM iodoacetamide (IAA) for 30 min. Finally, trypsin was added at a final concentration of 1:50 and left overnight at room temperature. Digestion was stopped by adding Trifluoroacetic acid (TFA) at a final concentration of 1%. The solutions containing peptides were then desalted using C18 tips (Zenati et al., 2023) and dried down using the Speed vac at 38 °C. For analysis, samples were reconstituted in a solution made of 0.1% formic acid (FA) and 2% acetonitrile (ACN).

### LC–MS/MS analysis

#### Proteomics profiling

Profiling was carried out using a Vanquish Neo chromatograph paired with an Orbitrap Exploris 480 mass spectrometer (Thermo Fisher Scientific, United States). For each sample, 10 μL was injected and separated along a 50 cm EasySpray C18 column maintained at 50 °C with a 60 min gradient. Solvent A was 0.1% formic acid in LC–MS grade water, and solvent B was 0.1% formic acid in 80% ACN. The column was first equilibrated to 4% solvent B at 600 nL/min before setting the flow rate to 250 nL/min. The gradient was increased from 5% of solvent B to 20% of solvent B over 30 min, then to 35% solvent B over 25 min. A final elution was carried out with 55% of solvent B over 1.5 min prior to stopping the MS acquisition and initiating a sequence of washing steps. Data-independent acquisition was performed in a positive mode. Full MS1 scans, from 380 to 985 m/z, were acquired every 3 s at 120,000 resolution, and MS2 scans were acquired from 145 to 1,450 m/z at 60,000 resolution.

#### Metabolomics profiling

For metabolomics, 10 μL was injected twice for each sample and eluted using a 30-min gradient as follows: 1% ACN was held for 2 min, ramping to 99% ACN over 15 min, held at 99% ACN for 3 min before re-equilibrating to 1% ACN for 10 min. Flow rates were 250 μL/min for elution and 350 μL/min for re-equilibration. The MS analysis was performed using a TimsTOF (Bruker, Darmstadt, Germany) with Apollo II electrospray ionization (ESI) source. The drying gas was set to flow at 10 L/min and the drying temperature to 220 °C and the nebulizer pressure to 2.2 bar. The capillary voltage was 4,500 V and the end plate offset 500 V. The peak intensities were log2 transformed, and only the metabolites present in at least 70% of the samples of at least one group were retained for statistical testing. The remaining missing values were imputed by half of the minimum value observed throughout the dataset.

### Data processing and statistical analysis

The raw files were first converted to mzML format using msconvert and processed using DIA-NN version 1.8.1 (Demichev et al., 2019). Spectral library was predicted using the Uniprot proteome for *S. aureus* (UP000054329, 2,937 entries, 14 September 2023). Precursors were filtered with a 1% false discovery rate (FDR) using a neural network classifier set to double-pass mode and set to 10 ppm precursor accuracy. The default trypsin/P enzymatic cleavage rule was used for *in silico* digestion and the MaxLFQ algorithm was used for label-free quantitation (LFQ) with a minimum of two shared peptides required. Protein hits to the target database were retained, while those matching to potential contaminants were removed prior to analysis. LFQ intensities were log_2_-transformed, and only those protein groups present in at least 70% of the samples of at least one group were retained for statistical testing. Significance was determined using Student’s *t*-test with *p* < 0.05 after correcting for multiple comparisons by the Benjamini-Hochberg FDR method. Analysis was performed in R using the packages “prcomp” for principal components analysis, “pheatmap2” for hierarchical clustering and heatmap visualizations, and “ggplot2” and “ggpubr” for protein abundance profiles. Gene set enrichment analysis (GSEA) was performed using “clusterProfiler” R package and significance was determined by a post-Benjamini-Hochberg adjusted *p* < 0.05. The data is deposited in the ProteomeXchange Consortium via the PRIDE (Proteomics Identifications Database), accession number (PXD071424).

Metabolomics data was processed using Metaboscape 4.0 (Bruker Daltonics, Billerica, MA, United States). The T-ReX 2D workflow was chosen and used following previously published settings (Zenati et al., 2023). Identification of metabolites was performed by matching the identified metabolites to the matching library. Metaboanalyst (Pang et al., 2024) was used to assess differential abundance metabolites (*p*-value < 0.05).

Receiver operating characteristic (ROC) curves and the corresponding area under the curve (AUC) were computed to evaluate the discriminatory performance of each candidate biomarker in distinguishing invasive from non-invasive MRSA strains. Analyses were performed in Python (v3.x) using the scikit-learn (v1.x) and matplotlib libraries.

## Results

### General and comparative proteomic analysis

**Figure 1:**
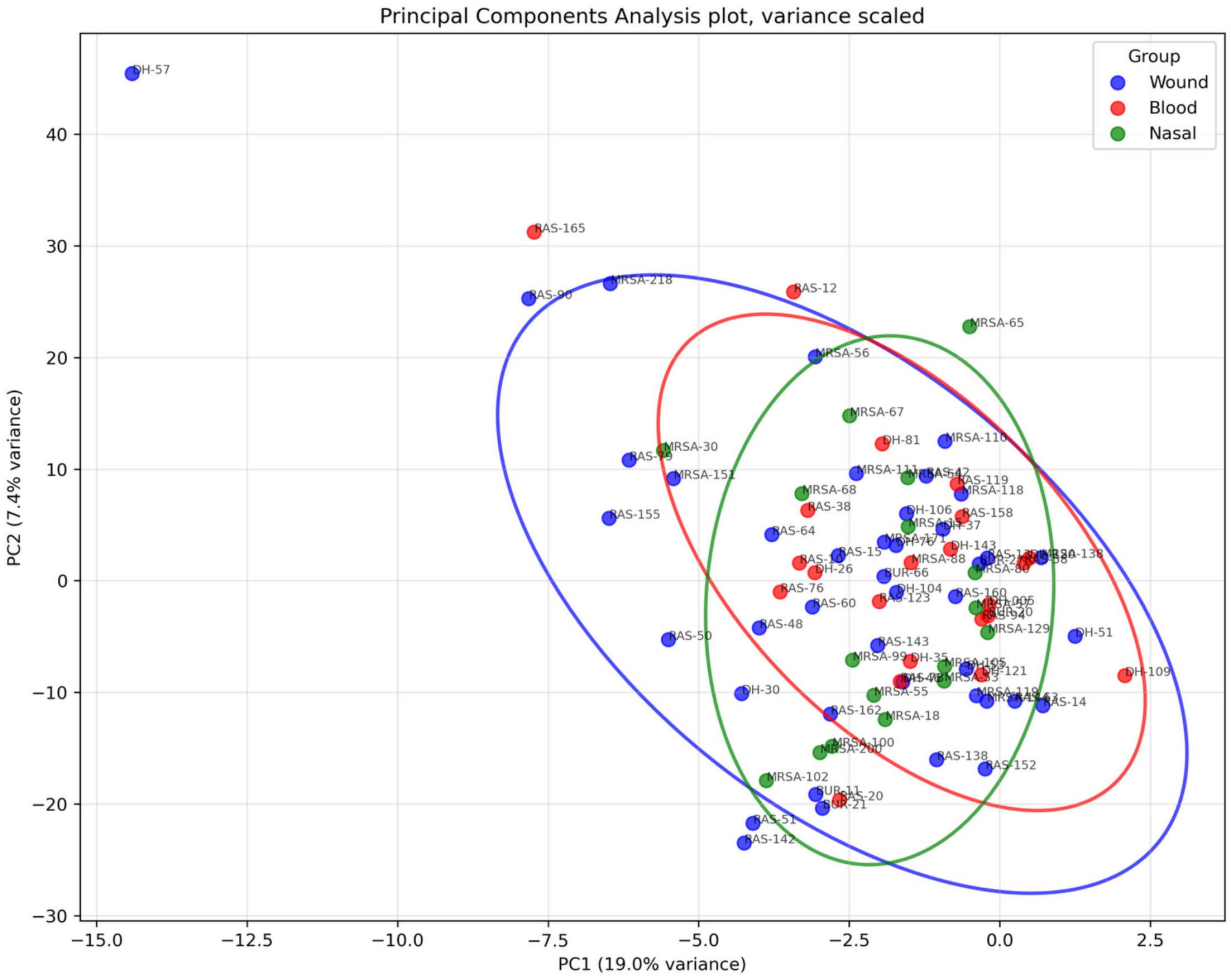
Principal component analysis (PCA) of proteomic profiles of MRSA isolates. Each point represents an isolate, color-coded according to clinical source (red = blood, green = nasal, blue = wound).

A total of 2,000 proteins were identified across all 90 isolates, with an average of 1,500 proteins identified per isolate. Comparison for the presence or absence of proteins between invasive and non-invasive isolates showed no distinct proteins being exclusively expressed in one group compared to the other. To explore the global proteomic variation across isolates, a principal component analysis (PCA) was performed on the LFQ intensities of all identified proteins. The PCA plot demonstrated no apparent clustering of isolates according to their clinical source (Figure 1). The first two principal components accounted for 19.0 and 7.4% of the variance, respectively, indicating that while source-specific differences exist, they represent only a modest proportion of the overall proteomic variance.

**Figure 2:**
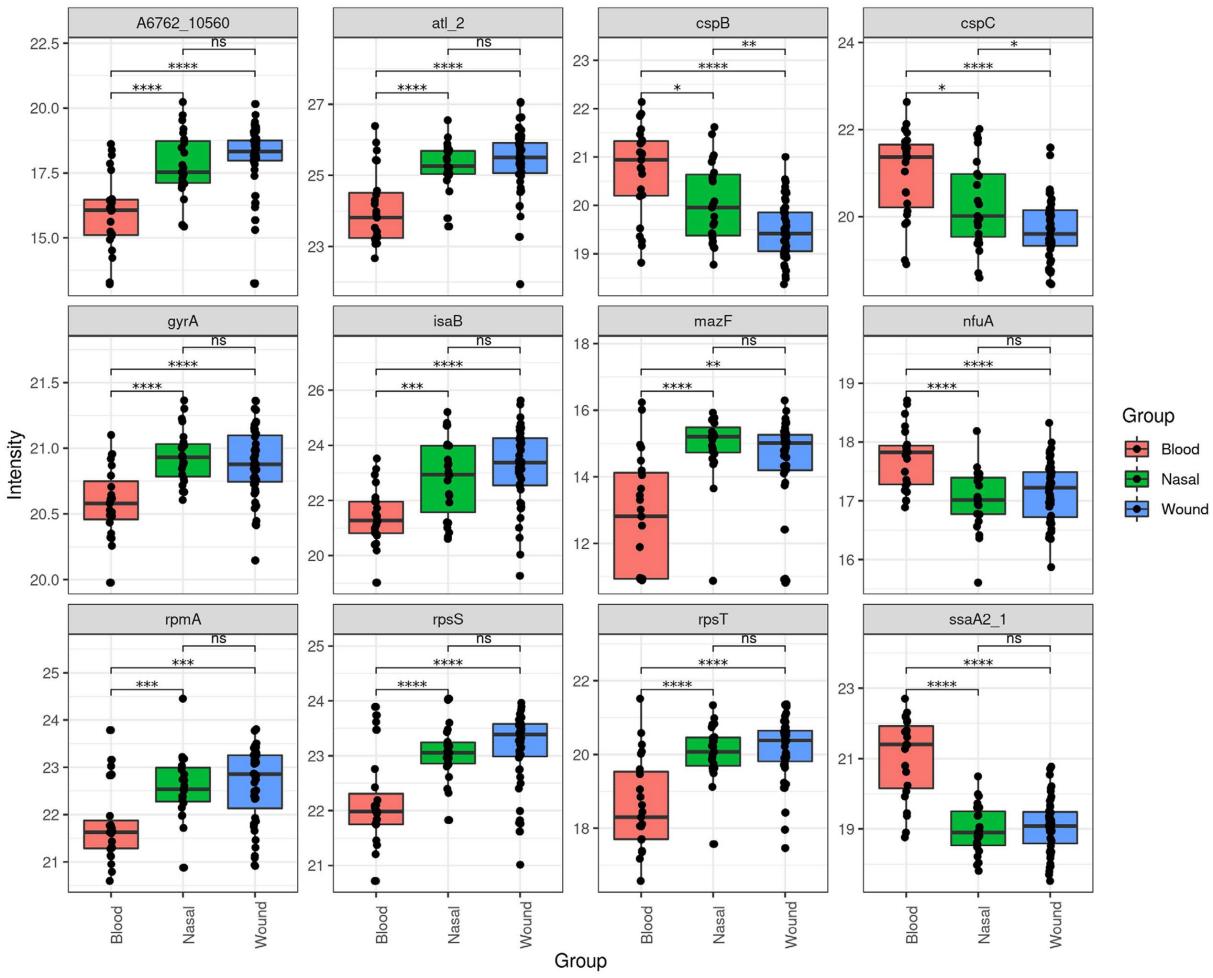
Top 12 most significant differential proteins abundance across MRSA isolates from blood, nasal, and wound sources.

Differential analysis showed one protein having a higher LFQ in invasive isolates when comparing them to non-invasive nasal isolates, and four proteins having higher LFQ in these non-invasive nasal isolates compared to invasive ones. Similarly, three proteins were recovered at higher LFQ in invasive isolates when comparing them to non-invasive wound isolates, while five proteins had higher LFQ in these wound isolates compared to invasive isolates. Among those proteins with differential LFQs, one protein (Staphylococcal secretory antigen SsaA2) was constantly recovered at a higher intensity in invasive isolates when comparing it to non-invasive (wound and nasal colonizer isolates). Moreover, two proteins (Small ribosomal subunit protein bS20 and Bifunctional autolysin atl_2) were recovered at higher intensity in non-invasive isolates when comparing them to invasive isolates (Figure 2).

### General and comparative metabolomic analysis

**Figure 3:**
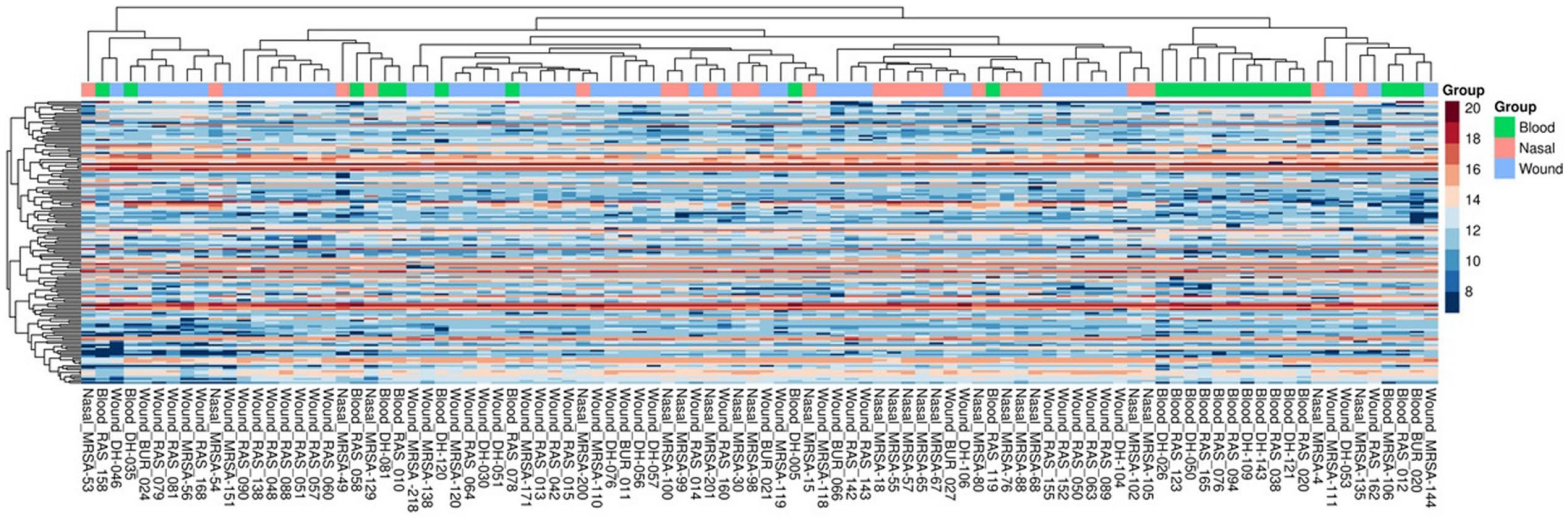
Heatmap of metabolomic profiles of MRSA isolates. Log₂-transformed expression values of metabolites are shown, with rows representing metabolites and columns representing isolates. The color scale indicates relative abundance (blue = lower, red = higher). Both metabolites and isolates were clustered using hierarchical clustering.

**Figure 4:**
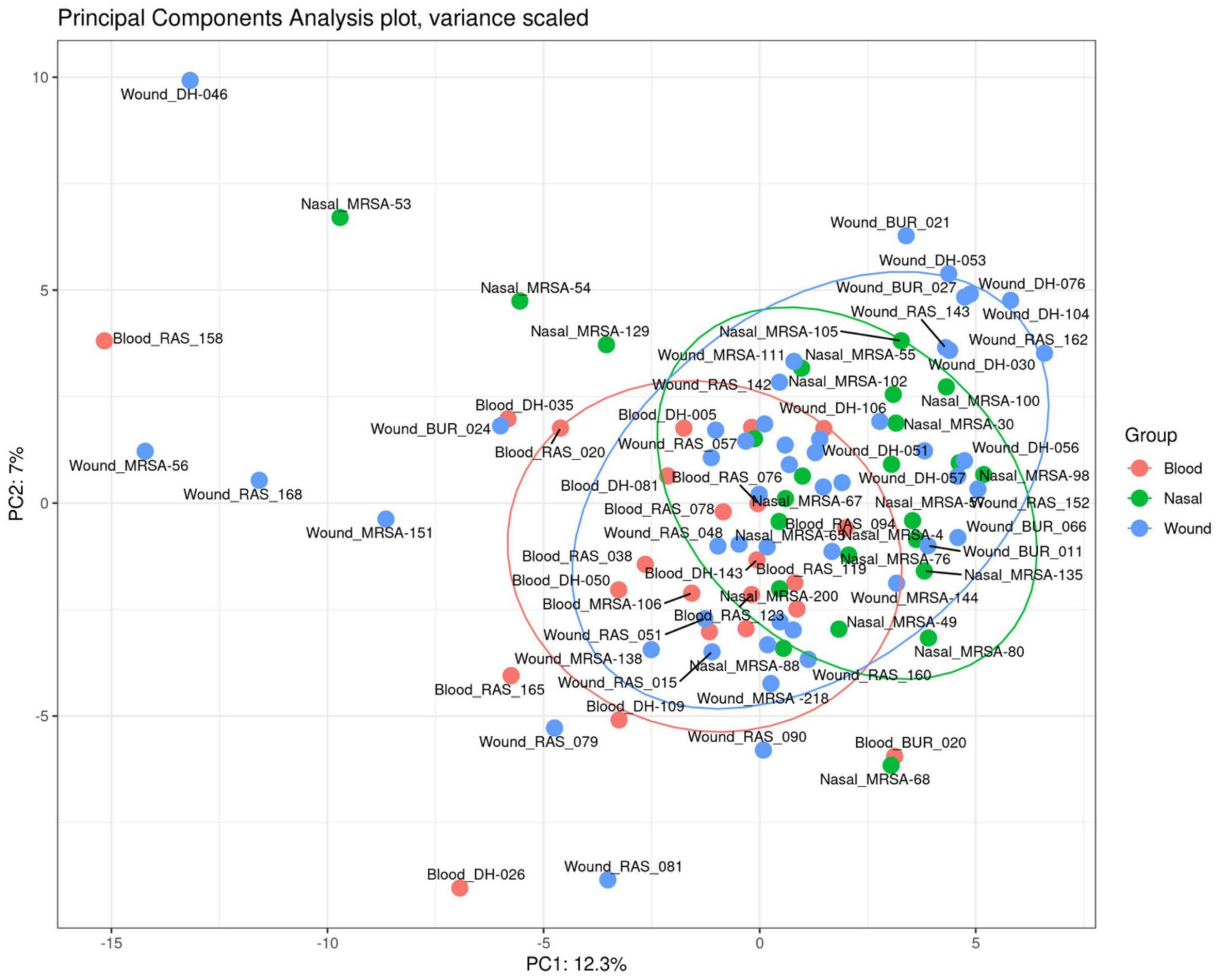
Principal component analysis (PCA) of metabolomic profiles of MRSA isolates. Each point represents an isolate, color-coded by clinical source (red = blood, green = nasal, blue = wound).

A total of 150 metabolites were identified across all isolates. All metabolites were identified across all three groups at varying intensities, with no unique metabolite identified (Figure 3). We next performed principal component analysis (PCA) on the metabolomic profiles to assess global clustering of isolates. The PCA plot revealed no clear separation between invasive blood isolates and non-invasive (wound and nasal colonizers) isolates (Figure 4). Instead, isolates from all three groups showed extensive overlap, consistent with the observation that metabolite identities were largely shared across them. The first two components explained 7 and 12.3% of the variance, respectively, highlighting that group-specific differences in metabolite abundances are subtle relative to the overall metabolic similarity among isolates.

**Figure 5:**
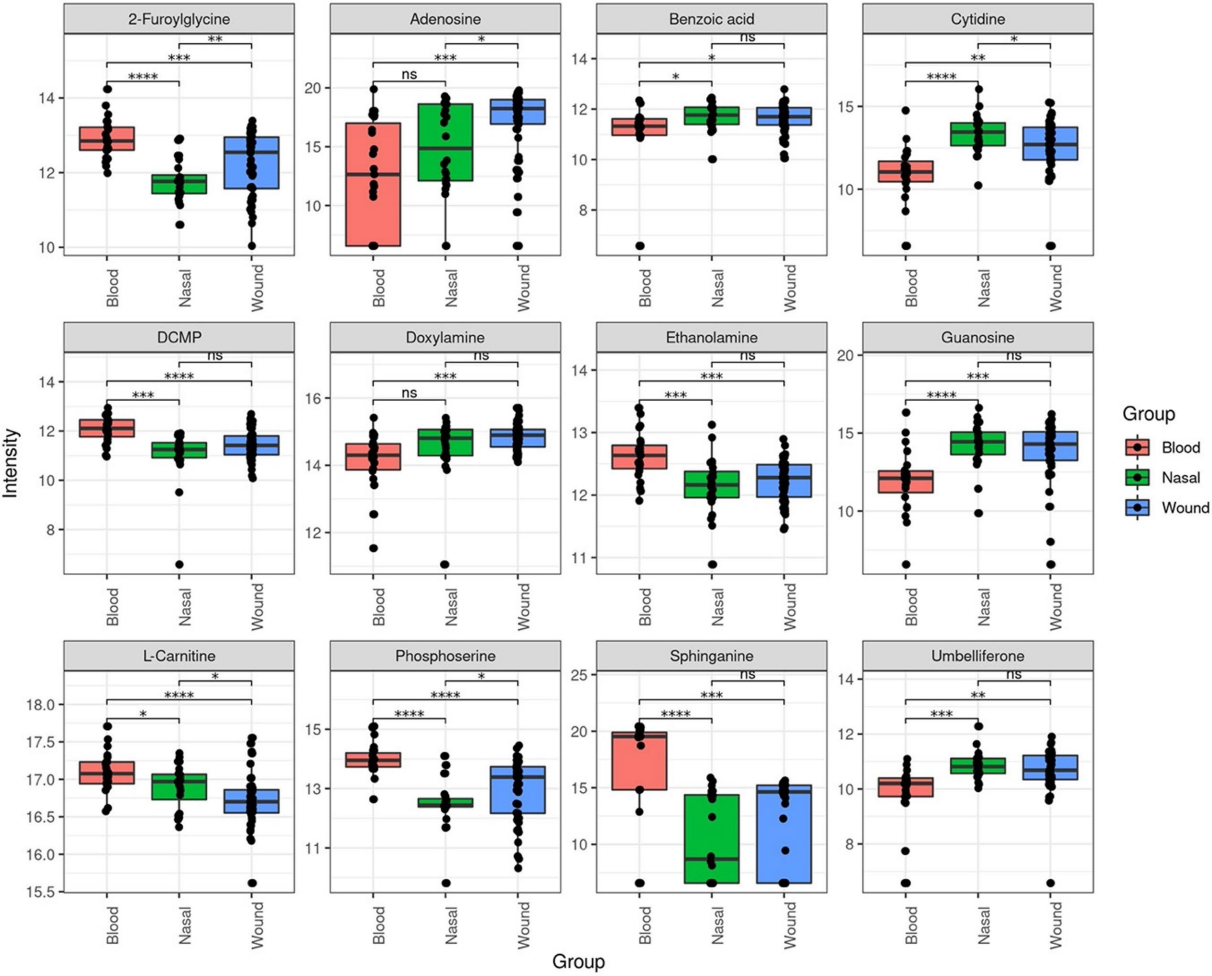
Top 12 most significant differential metabolite abundance across MRSA isolates from blood, nasal, and wound sources.

**Figure 6:**
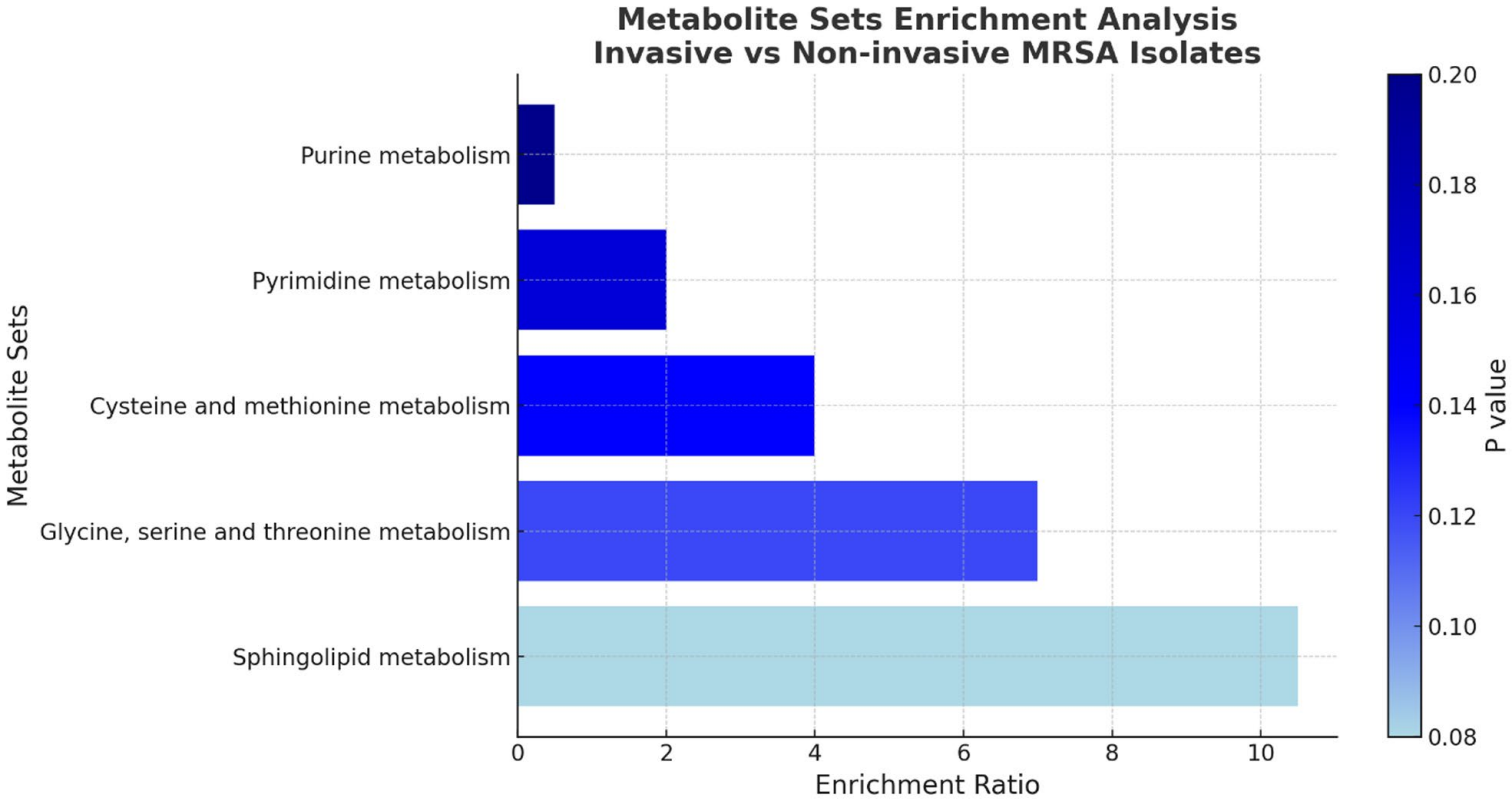
Enrichment analysis of pathways done on KEGG based on the metabolites identified with differential LFQ levels between invasive, non-invasive, and nasal colonizer isolates. The X-axis represents the level of enrichment of each pathway, and the color shows the level of significance for the enrichment.

When comparing non-invasive nasal colonizers vs. wound isolates, no significant differences were observed in metabolite intensities, suggesting that they shared broadly similar metabolic profiles. However, comparison of the nasal colonizers with invasive isolates revealed that 5 metabollites were recovered with higher intensity in invasive isolates, and 4 metabolites were recovered with higher intensity in the nasal colonizers. A similar trend was observed when comparing invasive isolates with the non-invasive wound isolates. Four metabolites were recovered at higher intensities in invasive isolates, while another four were more abundant in the wound isolates. Among the identified metabolites, two—sphinganine and phosphoserine—were consistently more abundant in invasive isolates compared to non-invasive nasal colonizers and wound isolates. Moreover, three metabolites—cytidine, benzoic acid, and guanosine—were found at lower intensities in invasive isolates relative to the nasal and wound isolates (Figure 5). These findings highlight distinct metabolic adaptations among isolates with different infection profiles. The observed metabolite variations may reflect bacterial strategies for survival, immune evasion, and adaptation. Enrichment analysis of KEGG pathways was performed to identify the pathways those metabolites belonged to. A total of 5 pathways were found to be enriched with the sphingolipid metabolism pathway being the most significantly enriched (Figure 6).

ROC curve analysis was performed for all identified candidate biomarkers along with the corresponding AUC values. The analysis showed that several biomarkers exhibited good discriminatory performance between invasive and non-invasive MRSA isolates. Among proteins, Atl (AUC = 0.797) and bS20 (rpsT) (AUC = 0.791) demonstrated good classification ability, while SsaA2 showed comparatively lower performance (AUC = 0.674). Among metabolites, phosphoserine exhibited the highest performance (AUC = 0.820), followed by cytidine (AUC = 0.801), guanosine (AUC = 0.789), sphinganine (AUC = 0.770), and benzoic acid (AUC = 0.761) (Supplementary Figure S1; Supplementary Table S1).

These findings support the potential of the identified biomarkers to distinguish invasive from non-invasive MRSA isolates.

## Discussion

Despite extensive research on phenotypic and molecular resistance profiles, comparative proteomic and metabolomic studies of MRSA strains from different clinical sources remain scarce (Asadollahi et al., 2018; Monecke et al., 2011; Senok et al., 2019, 2020; Boucherabine et al., 2025). Moreover, previous work done on MRSA has demonstrated the effect of various environmental conditions and stress on its proteomic profile and how those changes allow MRSA to adapt to the harsh environment and thrive in it (Resch et al., 2006; Liebeke et al., 2011; Liu et al., 2014; Atshan et al., 2015; Rahman et al., 2022; Sulaiman et al., 2021). Hence, this study focused on understanding the proteomics and metabolomics of invasive and non-invasive MRSA isolates to aid the elucidation of the bacterial factors affecting the success of isolates causing invasive disease.

Through the use of a previously established extraction method (Boucherabine et al., 2024), this study investigated the proteome and metabolome differences between invasive and non-invasive MRSA isolates. Indeed, the invasive and non-invasive MRSA isolates were shown to share highly conserved core proteome and metabolome, with only subtle quantitative differences between them. This aligns with accumulating evidence that colonizing strains can cause invasive infections without requiring major genetic or phenotypic changes (Räz et al., 2023). Räz et al. observed slightly reduced adherence capacity in invasive isolates compared to their patient-matched colonizing counterparts and this difference was mirrored by the lower abundance of the major autolysin Atl protein in the invasive isolates on proteomics profiling (Räz et al., 2023). Autolysin is a cell-wall hydrolase that also functions as an adhesin, it promotes bacterial attachment by facilitating surface protein display and biofilm formation. The absence of any protein or metabolite uniquely present in one group of our isolates is consistent with that study’s conclusion that no single “invasive factor” distinguishes invasive strains (Biswas et al., 2006; Leonard et al., 2023). Moreover, Autolysin Atl is a multifaceted virulence factor: it facilitates bacterial adhesion to host tissues (by binding extracellular matrix proteins like fibronectin) (Porayath et al., 2018) and modulates the release of other virulence determinants. For example, recent work demonstrated that Atl governs the post-translational sorting and secretion of the leukocidin toxin LukAB, thereby influencing cytotoxicity toward neutrophils (Zheng et al., 2022). In addition, Atl-driven autolysis can lead to secretion of cytosolic proteins; an Atl mutant was shown to secrete significantly fewer cytoplasmic proteins relative to wild-type, highlighting Atl’s key role in releasing immunomodulatory factors (Pasztor et al., 2010). Given these functions, the lower Atl expression in invasive MRSA could be advantageous during bloodstream infection by preventing excessive self-lysis and the unwarranted release of pro-inflammatory cell wall fragments creating a balance that avoids triggering host defenses too early. We hypothesize that invasive MRSA could have evolved to modulate Atl levels (and perhaps rely on alternative peptidoglycan hydrolases) to optimize survival in host tissues. Another protein that differed was the 30S ribosomal protein S20 (bS20), which we found at significantly lower levels in invasive isolates compared to non-invasive ones. bS20 is a component of the small ribosomal subunit and plays a role in ribosome assembly and mRNA binding during translation. Decreased abundance of a ribosomal protein suggests that invasive MRSA may adopt a translational or growth-rate shift to survive in hostile environment. Indeed, alterations in ribosomal protein expression and mutations in ribosomal genes have been linked to adaptive phenotypes in *S. aureus* (Mlynarczyk-Bonikowska et al., 2022).

The proteomic analysis also revealed that non-invasive MRSA isolates showed higher abundance of ribosomal proteins compared to invasive isolates. This finding implies that the non-invasive strains may maintain a higher translational capacity and biosynthetic activity, supporting growth and production of colonization factors in a less hostile environment. In contrast, invasive isolates exhibited a relative depletion of ribosomal components, suggestive of downregulation of protein synthesis as they shift into stress survival mode. This is in keeping with previously reported work which showed that *S. aureus* tends to enter a ribosome hibernation state to persist in harsh environments (Lipońska and Yap, 2021). Notably, our proteomic profiling also identified Staphylococcal secretory antigen SsaA2, which is an immunogenic antigen of *S. aureus*, as significantly elevated in invasive isolates. Prior studies have shown that SsaA2 is produced during human infection and is a target of the host immune response (Romero Pastrana et al., 2018), its heightened abundance in invasive MRSA may therefore indicate an adaption to the host environment, possibly contributing to immune evasion strategies.

Sphingolipid metabolism pathways have garnered significant attention due to the role they play in the pathogenesis of Sphinganine, a crucial intermediate in sphingolipid metabolism. It traditionally arises from sphingomyelin, a lipid found abundantly in host cell membranes (Mori et al., 2022). Despite the inability of *S. aureus* to independently synthesize complex sphingolipids, it may manipulate or utilize the host’s sphingolipid pathways to its advantage during pathogenic interactions (Kunz and Kozjak-Pavlovic, 2019). The enrichment of the sphingolipid pathway in the invasive isolates is of capacity for active engagement with host membrane lipids. Indeed, a study by Yan et al. noted that sphingolipid and fatty acid pathways are among the major host pathways affected upon *S. aureus* infection of macrophages (Yan et al., 2022). Conversely, the lower level of sphingolipid metabolism in non-invasive isolates could be due to the natural antibacterial activity of sphingosine, which was shown to damage the cell wall of *S. aureus* and inhibit bacterial growth (Arikawa et al., 2002). On the contrary, invasive strains, by actively lysing host cells and liberating sphingolipids, create a nutrient niche or modulate the immune response by having adapted mechanisms to withstand or exploit the resulting sphingolipid-rich environment (Kunz and Kozjak-Pavlovic, 2019). Metabolomic analysis provided further insights into the adaptive strategies of MRSA, highlighting tailored responses to the bloodstream milieu, possibly through the leverage of host lipids and adjustment of the purine/amino acid metabolism to thrive during invasive infection (Potter et al., 2020). This shift includes enhanced lipid and amino acid biosynthesis and a possible downregulation of growth-related metabolite pools – changes that likely help the bacteria adapt to the host environment. These metabolic adaptations mirror the proteomic changes which suggest that invasiveness in MRSA is accompanied by a broad physiological reprogramming.

Although differential protein and metabolite abundances were identified, functional validation through mechanistic assays is required to establish causal roles in invasiveness. Host factors, including immune responses and patient comorbidities, which could influence clinical outcomes, were beyond the scope of this analysis, and further work to assess these are recommended. In addition, although the ROC and AUC analysis was suggestive of their potential of identified proteins and metabolites as diagnostic biomarkers, the analysis was performed on the same dataset used for biomarker discovery. Therefore, the reported performance should be considered preliminary and requires validation in independent cohorts.

To conclude, our findings demonstrate that invasive and non-invasive MRSA isolates share a highly conserved proteome and metabolome, yet they differ in the quantitative regulation of specific proteins and metabolites, which highlight the physiological adaptations that optimize bacterial survival within the hostile environment of the bloodstream. These findings, although lacking the host factor, highlight candidate biomarkers and pathways of translational relevance, offering potential for the development of diagnostic tools and therapeutic targets.

## Data Availability

The datasets presented in this study can be found in online repositories. The names of the repository/repositories and accession number(s) can be found at: http://www.proteomexchange.org/, PXD071424.
