# Supplementary material for: Proteomic and metabolomic profiling of methicillin-resistant Staphylococcus aureus associated with invasive vs. non-invasive infections: uncovering key biomarkers and pathogenic pathways
Source: Front Microbiol. 2026 May 6;17:1798070. doi: 10.3389/fmicb.2026.1798070 (PMC13188211; doi:10.3389/fmicb.2026.1798070)

**Supplementary Figure1.** ROC curves of candidate protein and metabolite biomarkers for distinguishing invasive and non-invasive MRSA isolates. The area under the curve (AUC) values indicates the discriminatory performance of each biomarker


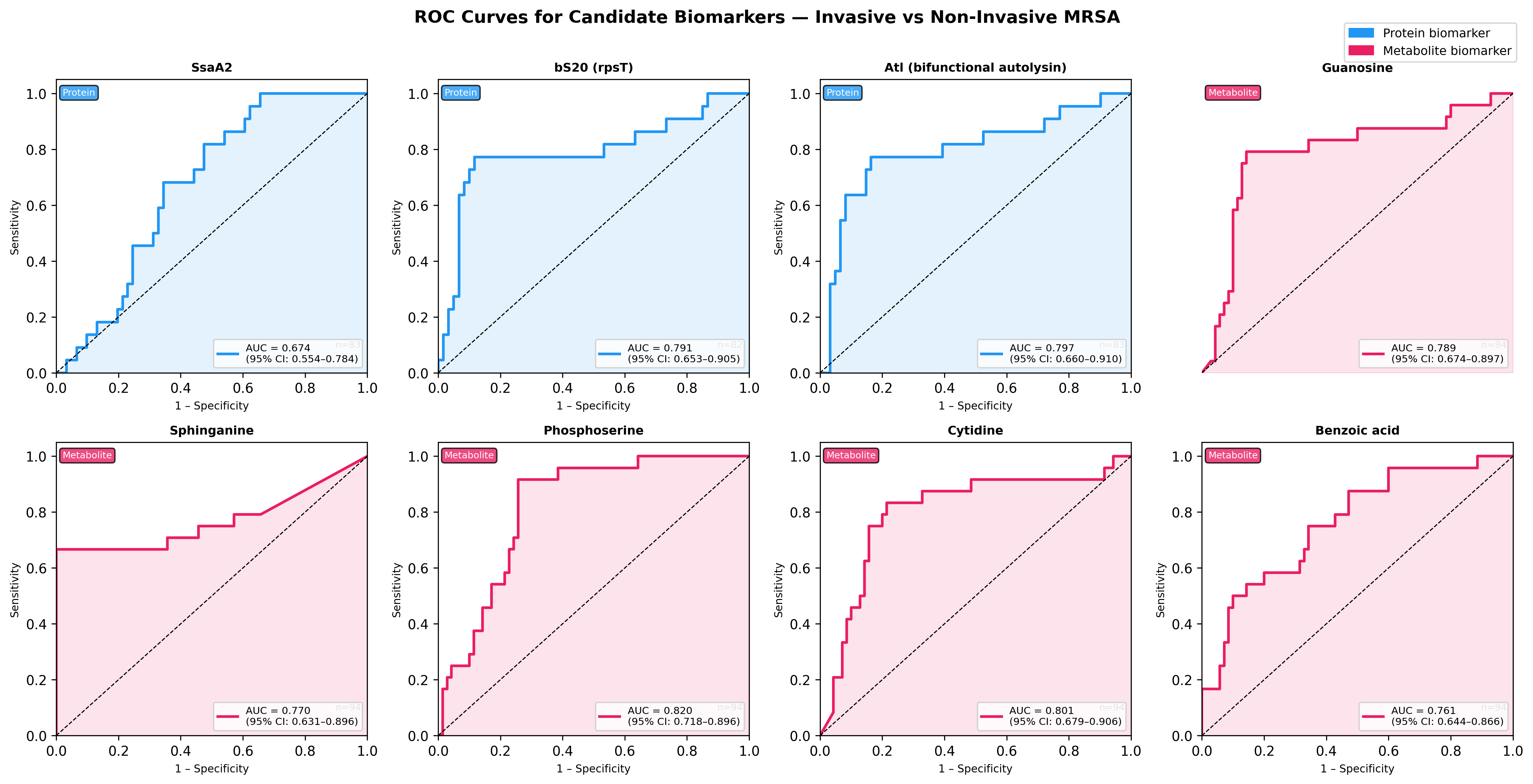

Supplement: Supplementary file 1 [file Supplementary_File_1.docx]
