# Supplementary material for: Proteomic and metabolomic profiling of methicillin-resistant Staphylococcus aureus associated with invasive vs. non-invasive infections: uncovering key biomarkers and pathogenic pathways
Source: Front Microbiol. 2026 May 6;17:1798070. doi: 10.3389/fmicb.2026.1798070 (PMC13188211; doi:10.3389/fmicb.2026.1798070)
Supplement: Supplementary file 2 [file Table_1.docx]

**Supplementary Table 1.** Biomarker performance table

| Biomarker | Type | AUC | Interpretation |
| --- | --- | --- | --- |
| SsaA2 | Protein | 0.674 | Moderate/Weak |
| bS20 (rpsT) | Protein | 0.791 | Good |
| Atl (autolysin) | Protein | 0.797 | Good |
| Guanosine | Metabolite | 0.789 | Good |
| Sphinganine | Metabolite | 0.77 | Acceptable–Good |
| Phosphoserine | Metabolite | 0.82 | Good–Strong |
| Cytidine | Metabolite | 0.801 | Good |
| Benzoic acid | Metabolite | 0.761 | Acceptable–Good |
